# Can Age or Height Define Appropriate Thresholds for Transition to Adult Seat Belts? An Analysis of Observed Seat Belt Fit in Children Aged 7–12 Years

**DOI:** 10.3390/ijerph19031524

**Published:** 2022-01-28

**Authors:** Anvay Parab, Tom Whyte, Bianca Albanese, Lynne Bilston, Sjaan Koppel, Judith L. Charlton, Jake Olivier, Lisa Keay, Julie Brown

**Affiliations:** 1 Neuroscience Research Australia, Sydney 2031, Australia; BAlbanese@georgeinstitute.org.au (B.A.); L.Bilston@neura.edu.au (L.B.); jbrown@georgeinstitute.org.au (J.B.); 2 The George Institute for Global Health, University of New South Wales, Sydney 2042, Australia; l.keay@unsw.edu.au; 3 Faculty of Medicine, University of New South Wales, Sydney 2052, Australia; 4 Monash University Accident Research Centre, Monash University, Melbourne 3800, Australia; sjaanie.koppel@monash.edu (S.K.); judith.charlton@monash.edu (J.L.C.); 5 School of Mathematics and Statistics, University of New South Wales, Sydney 2052, Australia; j.olivier@unsw.edu.au; 6 School of Optometry and Vision Science, University of New South Wales, Sydney 2033, Australia

**Keywords:** child occupant, seat belt fit, transition, anthropometry, vehicle geometry

## Abstract

This study aimed to investigate associations between demographic, anthropometric and vehicle factors and the fit of adult seat belts in children aged 7–12 years in passenger vehicles. Seat belt fit was assessed by inspection of 7–12-year-old children in their own cars. Logistic regressions examined associations between anthropometric and vehicle factors on achieving good seat belt fit. There were 40 participants included in the analysis, with 16 (40%) having good overall belt fit. The odds of achieving good overall seat belt fit increased by 15% (OR 1.15, 95% CI 1.04–1.27) with every centimeter increase in height and increased by 5% with every one-month increase in age (OR 1.045, 95% CI 1.001–1.10). Controlling for vehicle factors, neither age or height was significantly associated with overall good belt fit, and the discriminatory power of models including these metrics to predict good belt fit was 73% (AUC 0.73, 95% CI 0.55–0.91) and 74% (AUC 0.74, 95% CI 0.58–0.91). The results suggest that taller and older children have a better chance of achieving a good seat belt fit. However, with variations in seat geometry between vehicles, no single simple metric clearly defines an appropriate transition to the adult seat belt.

## 1. Introduction

The correct use of an appropriate restraint system is a highly effective way to protect children from death and serious injury in crashes [1,2,3]. Appropriate restraint use requires a restraint system that is the most appropriate for the size of the child. Different types of dedicated child restraints such as rearward-facing and forward-facing systems with integrated harnesses, and booster seats designed to be used with the vehicle seat belt system exist for young children. For children too large for booster seats, the only restraint available is the adult seat belt system. For optimum crash protection from an adult seat belt, the sash part of the seat belt should be positioned over the mid-portion of the clavicle, and lap portion positioned under the superior iliac spines of the bony pelvis [4,5,6,7,8]. Variations from this belt geometry have been shown to impair crash performance or to irritate the occupant and lead to them choosing an alternate, improper belt routing [2,5,6,8,9]. This seat belt position must also be maintained throughout the trip. For this to occur, the child should be able to naturally bend their knees around the front edge of the vehicle seat cushion to reduce the propensity for slouching and be able to comfortably maintain an upright posture with their back against the vehicle seat back. Appropriate restraint by an adult seat belt therefore requires an appropriate match between the child’s size and the geometry of the adult belt and vehicle seat.

In many countries, legislation controls when children transition from a dedicated child restraint system to an adult seat belt. In different countries and jurisdictions, different metrics and different thresholds for this transition are in place. For example, Australian regulations [10] require children not to use adult seat belts until they are at least 7 years old. Different states in the US variously require a child to be 6–9 years of age, 145 cm tall or 29–36 kg in weight before legally being able to use an adult seat belt [11]. Some European countries such as Germany, do not allow seat belt use until children are older than 12 years and/or taller than 150 cm [12]. This variation in metrics and thresholds demonstrates a lack of consensus around an optimal metric and threshold for defining appropriate transition from a dedicated child restraint or booster to an adult seat belt. While thresholds such as 145 [13] and 148 cm [14] are often discussed as appropriate thresholds, previous work measuring child anthropometry and/or vehicle seat and seat belt geometry indicate that good belt fit varies substantially based on child anthropometry and vehicle dimensions [14,15].

Rather than relying on a single metric or combination of metrics related to a child’s age, height, or weight, the “five-step test” is currently considered best practice in some jurisdictions for assessing the readiness of a child for transition to an adult seat belt. The five-step test compares various anatomical points of the child relative to the vehicle seat and seat belt, recognizing that vehicle seat dimensions and belt anchorage locations likely play a role in good belt fit for older children [16]. That is, the same child might achieve good belt seat belt fit in one vehicle, but poor belt fit in another. Standing height has been suggested to be suitably representative of the child’s anthropometry due to the relatively stable relationship between height and body segment lengths, although there remains considerable variation between individual children [17]. However, the transition to seat belts may not be able to be based on child anthropometry alone due to differences in vehicle seat and belt dimensions. Therefore, the aim of this study was to examine the relationship between child and vehicle characteristics and seat belt fit. Our hypothesis was that there is no single appropriate anthropometric cut-off above which all children achieve good belt fit.

## 2. Materials and Methods

### 2.1. Study Setting

This study uses data previously collected as part of a field-based observational study where participants were randomly recruited as they arrived at schools in the greater Sydney metropolitan region. One observation site was randomly selected from each of 5 randomly selected local government areas across the region. Potential participants in this larger observational study were parents/carers and their children aged 6–12 years. If more than one child aged 6–12 years was present in the vehicle, the child with the next birthday was included in the study. A total of 63 children aged 6–12 years, across the 5 data collection sites, participated.

### 2.2. Participants

For this analysis, data collected from all participants aged 7–12 years who were using an adult seat belt and for whom there was belt fit assessment data (*n* = 40) was extracted from the overall dataset of 63 children. Children aged 6 years were excluded from this analysis as children aged under 7 are required by law to use a booster or child restraint system. Participants observed with serious errors in seat belt use (e.g., “arms under seat belt”) were also excluded.

### 2.3. Data Collection

Data on type and quality of restraint use was collected by trained research assistants who observed and photographed children in their vehicles at the data collection site. The researcher also measured the standing height and weight of each child. The child’s demographics (e.g., age (in months) and gender) were obtained through a survey that was completed by the parent/carer. Observations made and documented included make and model of vehicle, seated position of the child and seat belt use and fit. Photos of the vehicles and children were taken with consent of the parent.

### 2.4. Seat Belt Fit

**Figure 1:**
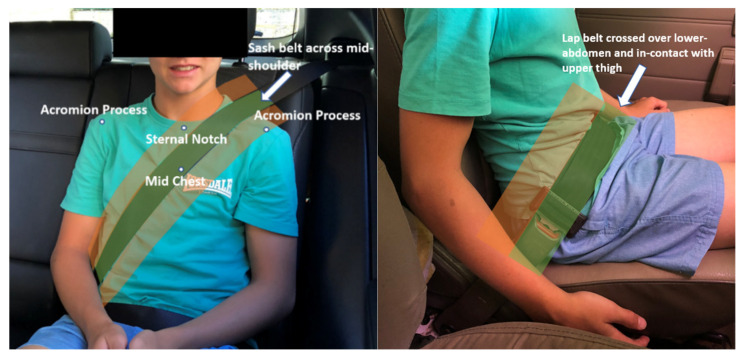
Photographs of a child passenger exhibiting good sash belt fit (sash across mid-shoulder), left, and good lap belt fit (lap belt crossed over lower-abdomen and in contact with upper thigh), right. The green shading indicates the belt position for a good fit and red shading indicates the belt position for a poor fit.

The primary outcome measure was quality of seat belt fit. Sash belt fit was defined as good if the sash passed over the mid portion of the shoulder, see Figure 1, and was otherwise coded as poor. Lap belt fit was defined as good when the lap belt passed over the lower abdomen with the bottom edge of the webbing in contact with the upper thigh, see Figure 1, and otherwise coded as poor. Overall good belt fit was defined as a participant having both good sash belt and lap belt fit. If one or both of sash belt fit and lap belt fit was coded as poor, overall belt fit was coded as poor. Assessments were made by the researchers on site and verified by at least two of the authors from photographs.

### 2.5. Other Variables

Age and gender were extracted from the parent survey for each child; and height and weight were extracted from on-site data collection forms. Vehicle make and model data was extracted from on-site observation records and verified from photographs. Make and model data was then used to categorize the vehicle type. Vehicles were categorized according to the Federal Chamber of Automotive Industries segmentation criteria. Vehicles fitting criteria for micro, light, and small passenger cars (vehicle footprint 8.3 m^3^ or less) were coded as small, and those fitting criteria for medium, large, upper large and people mover passenger vehicles (vehicle footprint greater than 8.3 m^3^) were coded as large. Seating position data was also extracted from the on-site observation data and categorized as front (for front passenger position) and rear (for any rear seat position).

### 2.6. Analysis

Univariate logistic regression analyses examined whether there was an association between age, gender, height, weight, vehicle type and seated location on each dependent variable of sash belt fit, lap belt fit and overall belt fit. Multivariable models were then used to examine the association between age, height and weight while controlling for vehicle factors. Given the expected correlation between age, height and weight (and potential for multicollinearity), and the small sample size, separate multivariable models were run for each of the anthropometric variables. Model fit and the predictive power of both the univariate and multivariable models were assessed using the Hosmer–Lemeshow test and the area under the receiver operating characteristic (ROC) curve (AUC with 95% Confidence Intervals), respectively. Interactions between variables were also explored. The probability of achieving good overall seat belt was determined from the regression results for anthropometric variables found to be significantly associated with belt fit, using the model (univariate or multivariable) with the best predictive power (i.e., largest AUC).

A collection of cases was extracted for further examination based on previous work that suggested that the minimum size child for using three-point belts alone is a stature of 148 cm and a weight of 37 kg [14]. This case series included children shorter than 148 cm and/or lighter than 37 kg who did achieve an overall good belt fit; and children 148 cm or taller and/or 37 kg or heavier who did not achieve an overall good belt fit. These cases were qualitatively examined to explore potential factors impacting seat belt fit.

## 3. Results

### 3.1. Sample Characteristics

Of the 63 participants in the available dataset, 22 were aged < 7 years, leaving a total of 41 children aged 7–12 years, all of whom were using adult seat belts. One of these children (height: 165 cm) was observed using the sash portion of the seat belt under their arm and thus, was excluded from the analysis as it indicated serious error and seat belt fit could not be determined.

Of the 40 children included, ages ranged from 7 to 12, with a mean age of 9.3 (SD 1.3). Almost two thirds of the participants (62%) were male. Measured standing height of the children ranged from 127 to 164 cm with a mean of 140.9 (SD 10.5). All children observed in the study were restrained appropriately according to current Australian legislation, 50% occupied the front passenger seat while the other 50% were seated in one of the rear seats of the vehicle. Almost two thirds of the children (62.5%) were travelling in large vehicles.

Twenty-four children (60%) met the criteria for good sash belt fit. Good lap belt fit was observed in 23 children (57.5%) while 16 (40%) had a poor lap belt fit. The quality of lap belt fit in one child was not able to be verified due to poor photo quality. Sixteen (40%) of the children met the criteria for good overall seat belt fit (i.e., good sash and lap belt fit), with 17 (42.5%) achieving either a good sash or lap belt fit, but not both. The remaining seven (17.5%) participants had neither a good sash nor lap belt fit.

### 3.2. Sash Belt Fit

Univariate logistic regression revealed a significant relationship between height and good sash belt fit. The odds of achieving a good sash belt good sash increased by 15% with every centimeter increase in height (OR 1.15, 95% CI 1.04, 1.27, AUC 0.82, 95% CI 0.68, 0.97), see Table 1. This association between good sash belt fit and increasing height remained when controlling for gender, seating position and vehicle size and there was little change in the estimate of the strength of this association (OR 1.16, 95% CI 1.03, 1.29, AUC 0.73, 95% CI 0.68, 0.97). There was no association between age or weight and sash belt fit even after controlling for gender, seating position or vehicle size.

### 3.3. Lap Belt Fit

There was no significant association between child anthropometry or vehicle factors and good lap belt fit in the univariate or multivariable models, see Table 2.

### 3.4. Overall Belt Fit

Univariate logistic regression revealed significant associations between both age and height with overall good seat belt fit. The odds of achieving a good overall seat belt fit increased by almost 5% with every 1-month increase in age (OR 1.05, 95% CI 1.00, 1.10, AUC 0.72, 95% CI 0.57, 0.88). For every 1-year (12-months) increase in age, the odds of achieving good seat belt fit increased by almost 80% (OR 1.77. 95% CI 1.01, 3.10). The odds of getting a good overall belt fit increased by about 8% for every centimeter increase in height (OR 1.09, 95% CI 1.00, 1.16, AUC 0.74, 95% CI 0.59, 0.90), see Table 3. However, the association of good overall belt fit with height and age was not significant in the multivariable models controlling for gender, seating position and vehicle size (AUC 0.74, 95% CI 0.58, 0.91 and 0.73, 95% CI 0.55, 0.91, respectively).

No significant interactions were identified in any of the multivariable models.

### 3.5. Probability Curves

**Figure 2:**
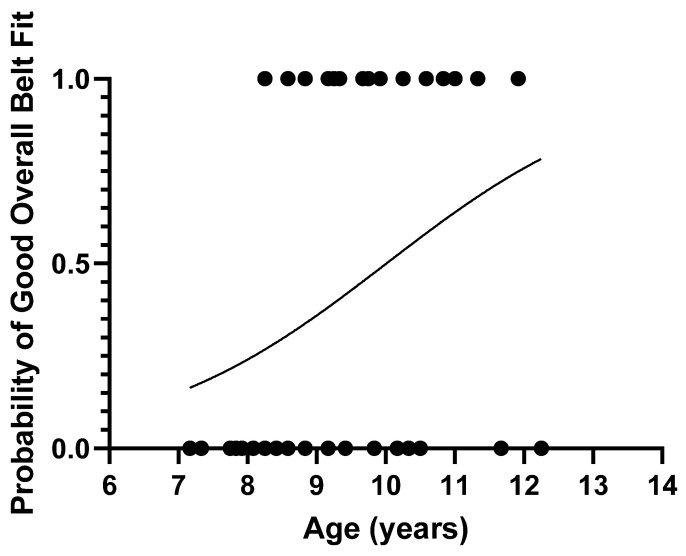
Probability curve for good overall seat belt fit by age of the child. Data points (circles) indicate ages of cases that achieved (1) and did not achieve (0) good overall seat belt fit.

The estimated probability of good overall belt fit using an age metric and results from the univariate regression is shown in Figure 2 and in brackets here for 7 years (15.1%), 8 years (24.0%), 9 years (36.0%), 10 years (49.9%), and 12 years (75.8%).

**Figure 3:**
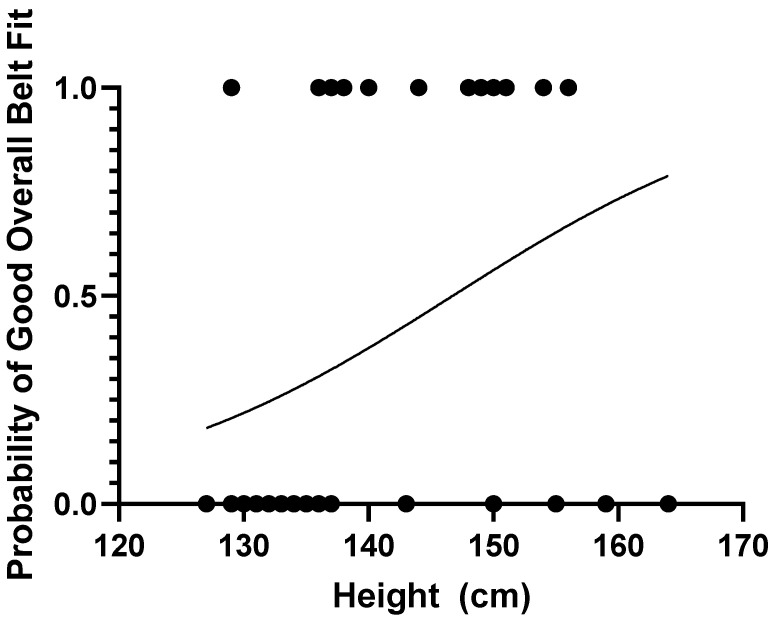
Probability curve for overall good seat belt fit by height of the child. Data points (circles) indicate heights of cases that achieved (1) and did not achieve (0) good overall seat belt fit.

The estimated probability of good overall belt fit using a height metric and results from the univariate regression is shown in Figure 3 and in brackets here for 135 cm (29.0%), 145 cm (46.6%), 148 cm (52.3%), and 150 cm (56.1%).

**Table 1:** Regression results for sash belt fit.

Variable	Ref.	Univariate				H-L χ^2^ (*p*)	Area under ROC (AUC)	Multivariable (with Height, H-L χ^2^ = 11.0, *p* = 0.139, AUC = 0.728, 95% CI 0.685, 0.975)				Multivariable (with Age, H-L χ^2^ = 7.081, *p*= 0.528, AUC = 0.714, 95% CI 0.536, 0.893)				Multivariable (with Weight, H-L χ^2^ = 4.895, *p* = 0.673, AUC = 0.702 95% CI 0.515, 0.890)			
		B	*p*	OR	95% CI		(95% CI)	B	*p*	OR	95% CI	B	*p*	OR	95% CI	B	*p*	OR	95% CI
Age (Months)		0.04	0.087	1.043	0.994, 1.094	6.584 (0.582)	0.686 (0.512, 0.860)					0	0.153	1.038	0.986, 1.093				
Gender	Female	0.45	0.506	1.571	0.414, 5.958	-	0.522 (0.369, 0.735)	0.45	0.655	1.57	0.215, 11.509	0.7	0.443	1.928	0.360, 10.320	0.75	0.376	2.117	0.403, 11.121
Height		0.14	0.005	1.15	1.043, 1.267	8.02 (0.432)	0.824 (0.681, 0.968)	0.14	0.01	1.16	1.034, 1.289								
Weight		0.05	0.163	1.049	0.981, 1.121	7.279 (0.403)	0.685 (0.505, 0.865)									0.042	0.293	1.043	0.965, 1.127
Seat	Front	0.42	0.519	1.519	0.425, 5.426	-	0.552 (0.368, 0.736)	−0.5	0.644	0.61	0.074, 5.006	0.3	0.714	1.362	0.261, 7.102	0.153	0.86	1.165	0.213, 6.381
Vehicle	Large	−0.6	0.448	0.545	0.114, 2.612	-	0.560 (0.365, 0.754)	−0.7	0.48	0.48	0.064, 3.650	−0.3	0.69	0.709	0.131, 3.847	−0.428	0.616	0.652	0.122, 3.477

Ref. = reference category; H-L = Hosmer–Lemeshow test; AUC = area under curve; CI = confidence interval.

**Table 2:** Regression results for lap belt fit.

Variable	Ref.	Univariate				H-L χ^2^ (*p*)	Areas under ROC (AUC)	Multivariable (with Height, H-L χ^2^ = 11.36, *p* = 0.184, AUC = 0.700, 95% CI 0.518, 0.882)				Multivariable (with Age, H-L χ^2^ = 8.959, *p* = 0.343, AUC = 0.704, 95% CI 0.521, 0.887)				Multivariable (with Weight, H-L χ^2^ = 5.491, *p* = 0.704, AUC = 0.645, 95% CI 0.458, 0.832)			
		B	*p*	OR	95% CI		(95% CI)	B	*p*	OR	95% CI	B	*p*	OR	95% CI	B	*p*	OR	95% CI
Age (Months)		0.03	0.206	1.03	0.984, 1.078	10.506 (0.231)	0.633 (0.445, 0.821)					0	0.444	1.019	0.971, 1.070				
Gender	Female	0.07	0.918	1.071	0.288, 3.985	-	0.508 (0.322, 0.695)	0.76	0.373	2.14	0.401, 11.430	0.9	0.309	2.45	0.432, 13.792	0.834	0.33	2.303	0.429, 12.349
Height		0.03	0.461	1.026	0.959, 1.097	7.441 (0.490)	0.586 (0.397, 0.774)	0.02	0.68	1.02	0.942, 1.096								
Weight		0.02	0.575	1.018	0.957, 1.082	3.803 (0.802)	0.557 (0.371, 0.743)									−0.006	0.868	0.994	0.924, 1.069
Seat	Front	0.95	0.155	2.593	0.697, 9.643	-	0.617 (0.436, 0.798)	1.07	0.21	2.92	0.546, 15.659	1	0.263	2.614	0.486, 14.054	1.213	0.174	3.364	0.586, 19.298
Vehicle	Large	−0.7	0.397	0.506	0.105, 2.442	-	0.568 (0.372, 0.764)	−0.4	0.617	0.66	0.125, 3.437	−0.2	0.785	0.792	0.148, 4.243	−0.398	0.639	0.672	0.128, 3.537

Ref. = reference category; H-L = Hosmer–Lemeshow test; AUC = area under curve; CI = confidence interval.

**Table 3:** Regression results for overall seat belt fit.

Variable	Ref.	Univariate				H-L χ^2^ (*p*)	Areas under ROC (AUC)	Multivariable (with Height, H-L χ^2^ = 6.436, *p* = 0.490, AUC =0.741, 95% CI 0.576, 0.907))				Multivariable (with Age, H-L χ^2^ = 7.686, *p* = 0.465, AUC =0.730, 95% CI 0.550, 0.911)				Multivariable (with Weight, H-L χ^2^ = 5.674, *p* = 0.578, AUC =0.689, 95% CI 0.504, 0.874)			
		B	*p*	OR	95% CI		(95% CI)	B	*p*	OR	95% CI	B	*p*	OR	95% CI	B	*p*	OR	95% CI
Age (Months)		0.05	0.047	1.049	1.001, 1.099	12.108 (0.146)	0.724 (0.566, 0.882)					0	0.137	1.039	0.988, 1.093				
Gender	Female	0.44	0.506	1.556	0.423, 5.721	-	0.522 (0.367, 0.737)	0.75	0.38	2.12	0.397, 11.338	1	0.254	2.62	0.501, 13.712	0.898	0.285	2.455	0.473, 12.737
Height		0.08	0.036	1.079	1.005, 1.158	9.596 (0.295)	0.740 (0.579, 0.900)	0.07	0.098	1.07	0.988, 1.158								
Weight		0.04	0.179	1.042	0.981, 1.108	4.588 (0.710)	0.658 (0.489, 0.826)									0.023	0.522	1.024	0.953, 1.100
Seat	Front	0.85	0.201	2.333	0.638, 8.538	-	0.604 (0.424, 0.758)	0.54	0.545	1.71	0.301, 9.690	0.6	0.461	1.882	0.350, 10.116	0.77	0.393	2.159	0.369, 12.624
Vehicle	Large	−0.6	0.447	0.563	0.127, 2.482	-	0.560 (0.361, 0.758)	−0.4	0.661	0.69	0.133, 3.604	−0.1	0.895	0.896	0.176, 4.562	−0.284	0.728	0.753	0.152, 3.736

Ref. = reference category; H-L = Hosmer–Lemeshow test; AUC = area under curve; CI = confidence interval.

### 3.6. Potential Enablers of Achieving Good Overall Seat Belt Fit

There were seven children shorter than 148 cm and/or lighter than 37 kg that achieved a good overall seat belt fit (see Table 4). Some factors that may have helped these children achieve a good sash belt fit identified through qualitative review of this data include sash belt upper anchorages positioned close to the top of the seat or the presence of sash belt height adjusters with adjustment to the lowest position. Factors that may have assisted achievement of good lap belt fit include short seat cushion lengths that allow the knees of the child to bend around the front edge without slouching, and outboard lap belt anchorages positioned forward of the seat bight.

### 3.7. Potential Obstacles to Achieving Good Overall Seat Belt Fit

There were five cases 148 cm or taller and/or 37 kg or heavier that did not achieve a good overall seat belt fit, see Table 5. Potential factors identified through qualitative review that may have contributed to these children not achieving a good overall seat belt fit include adjustable sash height anchorages positioned in the highest adjustment setting impacting sash belt fit, and lap belt anchorages at or behind the seat bight impacting lap belt fit.

## 4. Discussion

This study found that while there are increased odds of achieving a good overall seat belt fit with increasing age and height among children aged 7–12 years, neither are reliable as individual metrics to set a definitive threshold above which all children will achieve good belt fit in any car. This is clearly illustrated in the univariate and multivariable analyses where the predictive power of age and height for good overall belt fit was between 72 and 74% (based on AUC). Our analysis also estimates the probability of achieving good fit to be only 75% for the oldest children (12 years), and 56% for children with a height of 150 cm. Further, our case series identified circumstances where children who were shorter and lighter than commonly regulated cut-offs still achieved a good seat belt fit as well as instances where children taller and heavier than common cut-offs did not achieve a good fit, including the tallest and oldest children in the sample. This implies that factors other than child anthropometry likely contribute to seat belt fit. A shorter seat cushion length, adjustable and correctly positioned sash belt anchorages, and optimally located lap belt anchorages may enable better seat belt fit for child occupants.

According to legislation in New South Wales (NSW, Australia), a child may travel in an adult seat belt from 7 years of age [10]. This study showed that the probability of achieving a good seat belt fit at the age of 7 years is 15.1%. Safety recommendations often suggest a child needs to be at least 145 cm in height to achieve a good seat belt fit, and this is the threshold used in some states in the US [11]. The results from this study suggest that less than half (46.6%) of children will achieve good overall belt fit at this height (see Figure 3). Our results suggest that not all children will be appropriately restrained in an adult seat belt even with the most stringent thresholds, such as those used in Germany (aged > 12 years and >150 cm) [12]. In these cases, these children should remain in a booster seat since belt fit is generally improved through using a booster seat [14,15,18]. The results of this study indicate current thresholds regulating and recommending the transition to adult seat belts are poor predictors of good belt seat belt fit, and thus poor predictors of appropriate transition from booster seats to adult seat belts.

The current finding that vehicle-specific dimensions of the rear seat environment may contribute to belt fit confirms and builds on previous studies examining the compatibility between occupant size and vehicle dimensions [14,19]. For example, ‘slouching’ is a common response to a mismatch between thigh length and seat cushion length [14,19]. Slouching tends to cause the lap belt fit to be poor, due to angulation of the pelvis. This can arise due to vehicles having excessively deep seat cushions, and/or children having short thighs, since the important fit requirement is that the child’s thigh needs to be longer than the seat cushion depth of the vehicle for comfort, and to avoid slouching.

Other factors that may influence seat belt fit irrespective of the height and weight of the occupant are the locations of the seat belt anchorages. The height of the D-ring and its position with respect to the seat strongly influence the sash belt fit. Lap belt fit is affected by the angle of the lap belt (and hence the location of the lap belt anchorages) in combination with the cushion length [20,21,22]. Seat belt anchorage position varies from vehicle to vehicle. In our case series, we saw the majority of small children achieving good belt fit in vehicles where the lap belt anchorage was positioned in front of the seat bight, and there was an ability to adjust the sash height to a low position. However, the optimal position of these anchorages may vary depending on the individual child occupant’s anthropometry. It may be possible to define anthropometric thresholds for children to achieve a good fit in the adult seat belt based on vehicle seat and anchorage characteristics, or by vehicle make and model. However, further in-depth research is needed to examine whether specific vehicle features could predict good seat belt fit in combination with anthropometric thresholds, and/or whether recommendations could be made by vehicle make and model.

It is also important to note that there may be more nuanced anthropometric measures such as seated shoulder height and/or femur length that have stronger associations with good belt fit that were not examined in this study. This should also be explored in future research, although their practicality for ‘real world’ use is debatable. A limitation to keep in mind is that our findings regarding the potential influence of seat cushion length and upper anchorage locations were made on the observed geometry rather than measured geometry. However, as noted above these observations do align with previous work based on measured geometry. A further limitation is the sample size, which may not cover all children and vehicles. Additionally, we were not always able to identify all vehicle types retrospectively from the available data. Both of these factors may have impacted the power of the multivariable models to test significance of associations. While this study used observations of Australian children in vehicles available in Australia, the finding that a child’s age, height, and weight have limited power to predict good belt fit should be generalizable to other populations with similar vehicle mixes in the fleet. However, the probability curves presented are limited by the sample of observations (*n* = 40). Our observations on the potential relationship between belt anchorage characteristics were made through qualitative review of children beyond commonly used thresholds who failed to achieve good belt fit, and those below these thresholds who achieved good fit. There was no examination of the association between these factors and belt fit across the entire sample and further work is required to confirm the significance of these observances. A final limitation to note is that the observations were made when the vehicle was stationary, and seat belt fit may differ when the vehicle is in motion during a trip.

## 5. Conclusions

The results of this study suggest that there is unlikely to be an accurate, simple metric for defining thresholds for transition to adult seat belts that will ensure the vast majority of children using adult belts are optimally protected in all cars. This indicates that advice such as the five-step test that asks parents to consider the actual fit of their child in the seat and seat belt of their specific vehicle represents the best practice currently available.

## Figures and Tables

**Table 4:** Demographic and vehicle factors for cases shorter and lighter than cut-offs, who achieved a good seat belt fit.

Age Group	9–10 Years						
Gender	Female	Male	Female	Male	Male	Male	Male
**Height (cm)**	129	136	136	137	138	140	144
**Weight (kg)**	26	35	40	28	30	31	36
**BMI**	15.6	18.9	21.6	14.9	15.8	15.8	17.4
**Vehicle Size**	Small	UNK	Medium	UNK	Medium	Medium	Small
**Seated Position**	Centre Rear	Front	Outboard Rear	Outboard Rear	Outboard Rear	Front	Front
**Upper Sash Belt Anchorage Position**	Low	Low	High and behind seat back	High and behind seat back	High and front of seat back	High	Adjustable
**Knee Position Relative to Cushion**	UNK	UNK	Knees at cushion edge	Knees at cushion edge	UNK	Knees beyond cushion length	UNK
**Observed Slouch**	UNK	UNK	Slight	Minimal	UNK	UNK	UNK
**Lap Belt Anchorage Position**	UNK	UNK	Forward of seat bight	Forward of seat bight	Forward of seat bight	On floor behind seat bight	UNK

UNK = unknown, due to unavailability of information and inability to verify in available photographs.

**Table 5:** Demographic and vehicle factors for cases taller and heavier than cut-offs, who did not achieve a good seat belt fit.

Age Group	10–12 Years				
**Gender**	Male	Male	Male	Male	Male
**Height (cm)**	150	150	155	159	164
**Weight (kg)**	43	65	43	39	56
**BMI**	19.1	28.9	17.9	15.4	20.8
**Vehicle Size**	Medium	Light	Medium	Small	Medium
**Seated Position**	Rear Centre	Front	Front	Rear Outboard	Front
**Upper Sash Belt Anchorage Position**	UNK	High	UNK	High and behind seat back	Low
**Knee Position Relative to Cushion**	Knees beyond cushion length	Knees beyond cushion length	Knees beyond cushion length	Knees beyond cushion length	UNK
**Observed Slouch**	Minimal	UNK	UNK	None	None
**Lap Belt Anchorage Position Relative to Seat Bight**	Behind seat bight	Unknown	Unknown	At seat bight	Behind seat bight
**Sash Belt Fit**	Good	Poor (contacting neck)	Poor (contacting neck)	Good	Good
**Lap Belt Fit**	Poor (high)	Poor (high)	Good	Poor (high)	Poor (high)

UNK = unknown, due to unavailability of information and inability to verify in available photographs.

## Data Availability

The data presented in this study are available on request from the corresponding author.
